# ELV-N32 and RvD6 isomer decrease pro-inflammatory cytokines, senescence programming, ACE2 and SARS-CoV-2-spike protein RBD binding in injured cornea

**DOI:** 10.1038/s41598-021-92293-x

**Published:** 2021-06-17

**Authors:** Thang L. Pham, Jiucheng He, Azucena H. Kakazu, Jorgelina Calandria, Khanh V. Do, Robert Nshimiyimana, Ting F. Lam, Nicos A. Petasis, Haydee E. P. Bazan, Nicolas G. Bazan

**Affiliations:** 1 Neuroscience Center of Excellence, School of Medicine, Louisiana State University Health New Orleans, 2020 Gravier St., Ste. D, New Orleans, LA 70112-2223 USA; 2 Department of Chemistry and Loker Hydrocarbon Research Institute, University of Southern California, Los Angeles, CA USA

**Keywords:** Drug discovery, SARS-CoV-2

## Abstract

The severe acute respiratory syndrome coronavirus 2 (SARS-CoV-2) infection that causes coronavirus disease 2019 (COVID-19) has resulted in a pandemic affecting the most vulnerable in society, triggering a public health crisis and economic collapse around the world. Effective treatments to mitigate this viral infection are needed. Since the eye is a route of virus entrance, we use an in vivo rat model of corneal inflammation as well as human corneal epithelial cells (HCEC) in culture challenged with IFNγ as models of the eye surface to study this issue. We explore ways to block the receptor-binding domain (RBD) of SARS-CoV-2 Spike (S) protein to angiotensin-converting enzyme 2 (ACE2). We found that the lipid mediators, elovanoid (ELV)-N32 or Resolvin D6-isomer (RvD6i) decreased the expression of the ACE2 receptor, furin, and integrins in damaged corneas or IFNγ-stimulated HCEC. There was also a concomitant decrease in the binding of Spike RBD with the lipid treatments. Using RNA-seq analysis, we uncovered that the lipid mediators also attenuated the expression of pro-inflammatoy cytokines participating in hyper-inflammation and senescence programming. Thus, the bioactivity of these lipid mediators will contribute to open therapeutic avenues to counteract virus attachment and entrance to the body.

## Introduction

In December 2019, a new infectious respiratory disease (Coronavirus disease 2019, COVID-19^1^) caused by severe acute respiratory syndrome coronavirus 2 (SARS-CoV-2) emerged^2,3^, quickly becoming a pandemic and a global threat to public health. The virus has a single-stranded RNA with a 30 kb genome that encodes the Spike (S) protein containing a receptor-binding domain (RBD) for the angiotensin-converting enzyme 2 (ACE2) receptor^4^. Furthermore, the S protein contains cleavage sites for proteases FURIN and transmembrane serine protease 2 (TMPRSS2) that allow viral cell entrance^5^. Cells from the eye surface, lung alveoli, GI tract, among others, co-expressed *Ace2* and *Tmprss2* genes^6^.

The eye surface is a route of SARS-CoV-2 entrance^7,8^. One of the consequences of this viral infection is the inflammatory response in affected individuals, particularly in the aging population. Some lipid mediators that modulate inflammatory responses have been hypothesized to counteract COVID-19 pathology^9–11^. Lipid mediators facilitate debris clearance and antagonize pro-inflammatory cytokines by fostering inflammation resolution^12,13^. Here, we study lipoxin A4 (LXA4) derived from ω-6 arachidonic acid^14^, the R,R stereoisomer of Neuroprotectin D1 (NPD1)^15,16^, and Resolvin D6-isomer (RvD6i)^17^ derived from ω-3 docosahexaenoic acid (DHA), and Elovanoid (ELV)-N32. RvD6i and ELV-N32 are new lipid mediators discovered in our laboratory. Elovanoids are named because they are derived from very long chain polyunsaturated fatty acid products of the Elongation of Very Long Chain fatty acids protein 4 (ELOVL4) enzyme^18,19^ with pro-homeostatic and neuroprotective bioactivity^13,17,18^. Here, we show that ELV-N32 and RvD6i selectively decrease the ACE2 receptor expression and S protein RBD binding to the cornea stroma in an in vivo rat model of eye injury. We confirm that *Ace2* is an interferon-stimulated gene in human corneal epithelial cells (HCEC), a mechanism that would enhance the infectivity of SARS-CoV-2^20^. Therefore, we use HCEC in culture challenged with IFNγ to demonstrate that ELV-N32 or RvD6i exert blockage of ACE2 expression and RBD binding, hyper-inflammation, senescence programming, and pro-inflammatory cytokines.

## Results

### Lipid mediators decrease cornea injury-induced expression of ACE2 and binding of spike protein RBD

**Figure 1:**
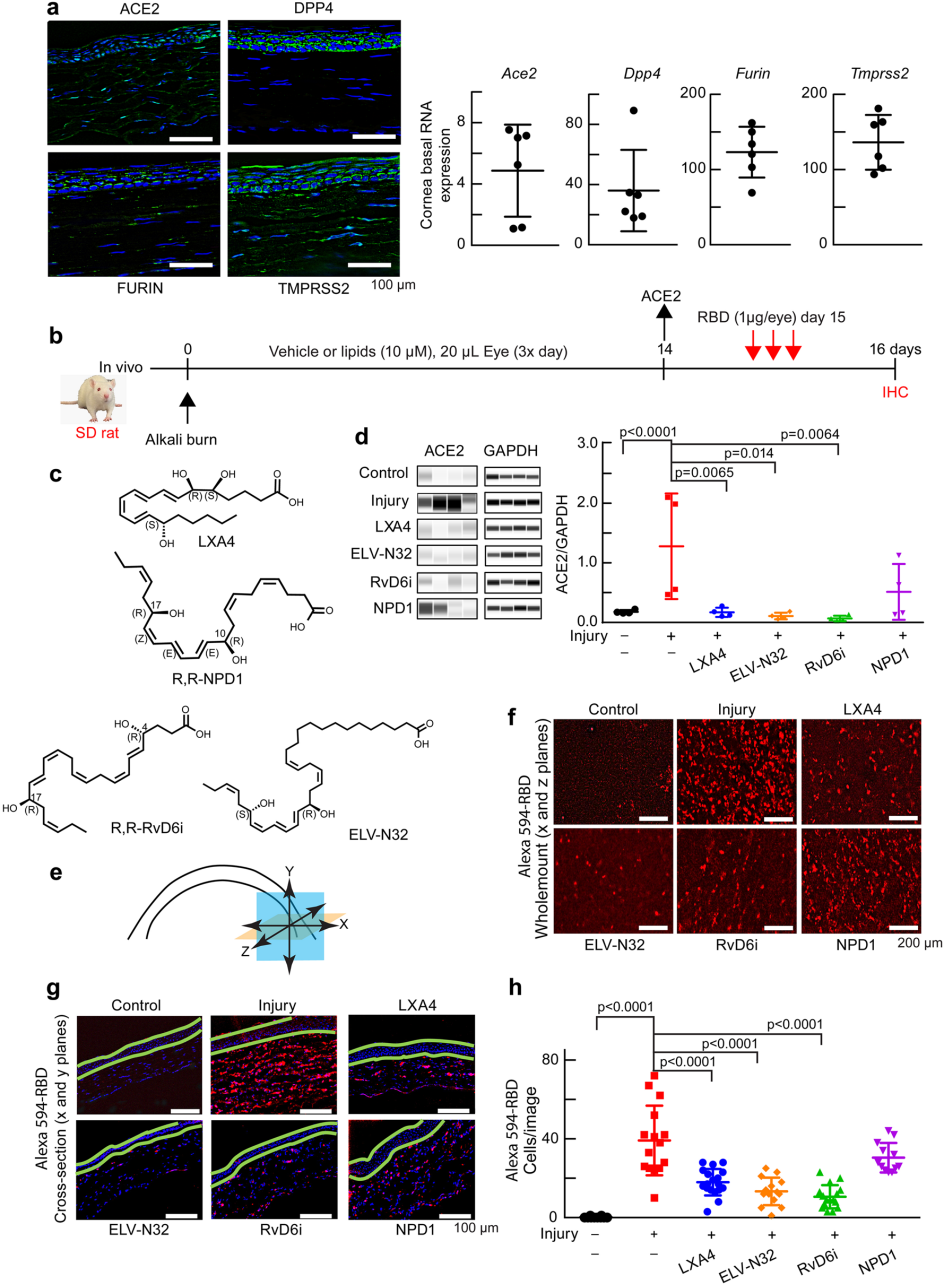
Selective lipid mediators reduce cornea injury-induced expression of ACE2 and binding of Alexa 594-RBD. (**a**) Expression of *Ace2*, *Dpp4*, *furin* and T*mprss2* in the uninjured rat cornea. Left: representative immunofluorescence imaging. DAPI stains nuclei (blue). Immunofluorescence shows ACE2 expressed in the epithelium and stroma. Right: RNA-seq data. (**b**) Experimental design. After alkali burn, rats received eye drops of lipid mediators or vehicle 20 μL/eye, 3 times/day for 14 days (double-blinded). ACE2 expression was assayed at day 14 after injury ± lipids treatment. At day 15, rats were treated with Alexa 594-RBD (1 µg/eye, 3 times) and corneas examined a day later. (**c**) Lipid mediators studied. The chirality in all figures of RvD6i and NPD1 used in this study had the R,R stereochemistry. (**d**) ACE2 abundance before and after injury ± lipids using Jess capillary-based Western Blot system (Protein Simple). ACE2 densitometry normalized to GAPDH in the same capillary to minimize errors. Data is from one rat cornea for each data point (N = 4). The p-values of ANOVA-post hoc Dunnett's multiple comparisons test with vehicle as reference are shown. Mean and SD are depicted as the lines. (**e**) Illustration showing corneal analysis by wholemount (x and z planes—orange color) and cross-section (x and y planes—blue color). (**f**) Wholemount images of binding of Alexa 594-RBD in corneas after injury and treatments. The control cornea (no-injury) has very low Alexa 594-RBD signal, while the injured cornea shows intense fluorescence. LXA4, ELV-N32, and RvD6i decrease Alexa 594-RBD binding while NPD1 fails. (**g**) Cross-section images of the same corneas shown in (**f**). The green lines were added to separate the epithelium from the stroma. Most of Alexa 594-RBD signal was found in the stroma. (**h**) Quantification of Alexa 594-RBD positive cells. Each data point represents number of cells/cross-section image. Values are means ± SD and p-values calculated by ANOVA-post hoc Dunnett's multiple comparisons test with vehicle as reference (4 images/cornea and 4 rat corneas/condition). The map of image capture is shown in Supplementary Fig. S2.

The SARS-CoV-2 receptors, including ACE2 and DPP4^5^, and the host proteases for the S protein, including FURIN^6^ and TMPRSS2, are expressed in the cornea (Fig. 1a), indicating that it is a potential site for SARS-CoV-2 entrance, in agreement with clinical studies showing infected patients’ epiphora, conjunctival congestion, or chemosis^21^. SARS-CoV-2 triggers lung injury and a systemic dysfunction of the inflammatory-immune system reflected in the cytokine storm^22,23^. We found that our cornea injury model recapitulates inflammatory-immune system dysfuctions^24^, including ACE2 receptor expression upon injury. To identify mediators that modulate these responses and to understand consequent mechanisms, we tested the following lipid mediators: LXA4, ELV-N32, RvD6i, and NPD1 (Fig. 1b,c). LXA4, ELV-N32, and RvD6i decrease ACE2 abundance and gene expression levels (Supplementary Fig. S1) to non-injured tissue, while NPD1 shows no consistent effect (Fig. 1d and Supplementary Fig. S1). Alexa 594-RBD displayed remarkable binding to the injured corneal stroma, and LXA4, ELV-N32, and RvD6i counteracted these injury-induced effects; however, NPD1 did not (Fig. 1e–h). Thus, there is a correlation between changes in the ACE2 receptor and RBD binding in the cornea after injury and lipid treatment. Although NPD1 decreased Dpp4 expression (Supplementary Fig. S1), it failed to inhibit the binding of the RBD to the damaged cornea. In addition, a recent publication reported that the SARS-CoV-2 virus could not enter cells expressing DPP4 as efficiently as cells expressing ACE2^4^. Therefore, we suggested that the RBD binding of the S protein was to ACE2 rather than to DPP4. Interestingly, most of the RBD was detected in the stroma and neutrophils, and inflammatory cells labeled with CD68 showed co-localization with RBD (Supplementary Fig. S2b). The population of these two cell types is increased in the cornea after injury and diminished by the lipid mediator treatments (Supplementary Fig. S2c).

### Lipid mediators disrupt the ACE2 upregulation, hyper-inflammation, senescence programming, and pro-inflammatory cytokines in the injured cornea in vivo

**Figure 2:**
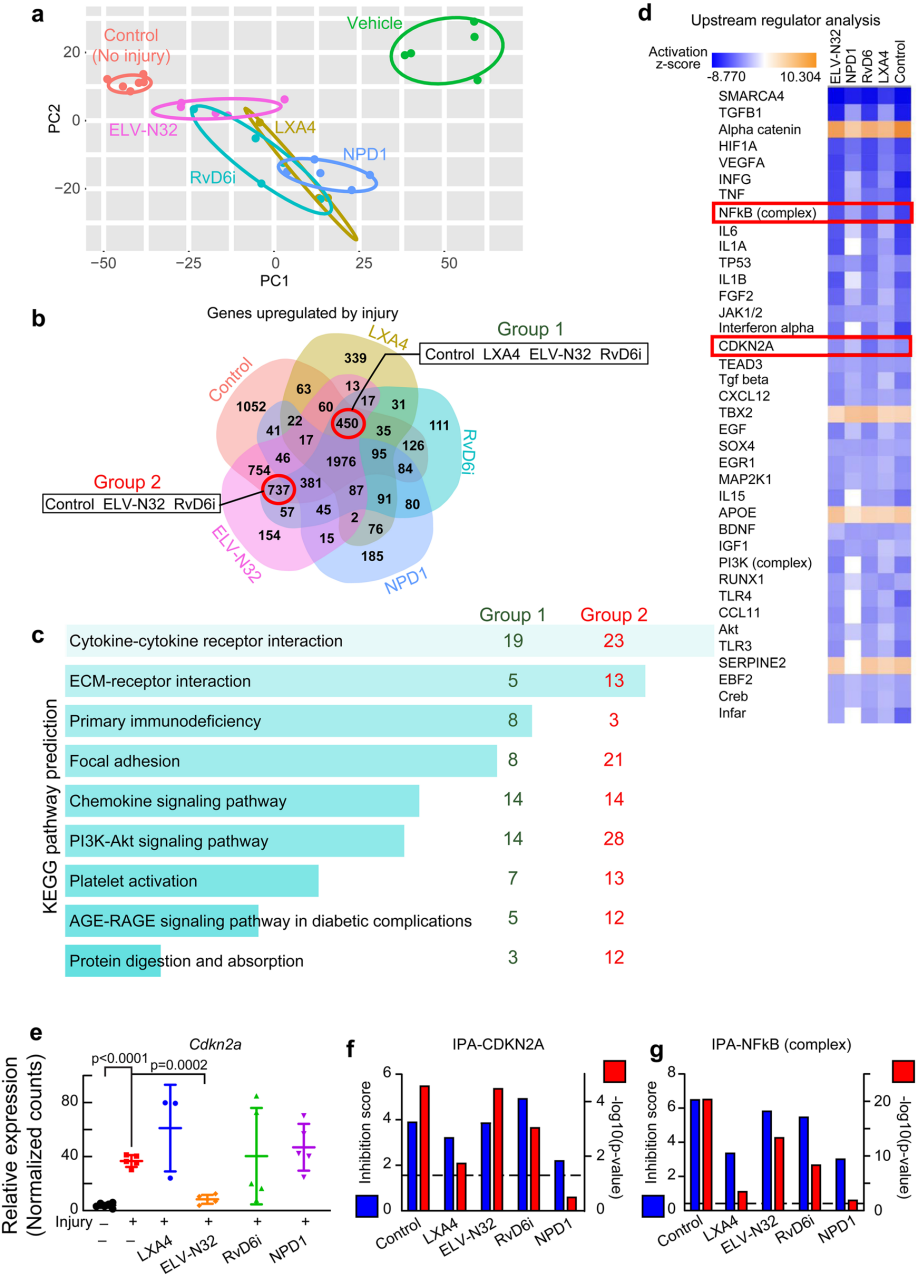
Selective lipid mediators disrupt ACE2 upregulation and injury-mediated hyper-inflammation, senescence, and pro-inflammatory cytokines components. (**a**) PCA plot of RNA-seq data. Rat corneas were analyzed at day 14 after injury ± treatments (Fig. 1b). Each data point represents one animal (N = 5/group, except LXA4 with N = 3 and control with N = 6). The eclipse of 95% confidence interval was used to group data points from the same set of treatment. (**b**) Venn diagram of significant genes (FDR < 0.05) upregulated by the vehicle treatment of injured corneas (RNA-seq data set was analyzed using DEseq2 with vehicle injured corneas as reference). The negative log2 fold change genes (upregulated by vehicle) with FDR < 0.05 were used. We excluded NPD1 because it failed to decrease *Ace2* expression upon injury (Fig. 1d). The groups of shared genes between control-LXA4-ELV-N32-RvD6i and control-ELV-N32-RvD6i are depicted in red circles. (**c**) The KEGG-pathway enrichment networks obtained from EnrichR tool of selected genes from (**b**). Bars were sorted by p-value. The length of the bar represents the significance of the pathway, while the lighter the color, the higher the significance. The number shows amount of genes from denoted group that are enriched in each pathway. (**d**) IPA upstream regulator analysis of significant genes vs. vehicle (injury) group. There are proteins with negative activation z-score compared to vehicle group (blue color). Among those are CDKN2A and NFkB (complex). (**e**) RNA-seq normalized counts of *Cdkn2a* gene that encodes the senescence key-marker p16INK4a; ELV-N32 decrease its expression. Data correspond to one cornea for each data point and is presented as mean ± SD. The p-values were analyzed by ANOVA-post hoc Dunnett's multiple comparisons test with vehicle as reference. The normalized counts were used for analysis. IPA scores for CDKN2A (**f**) and NFkB (complex) (**g**) upstream regulators. The left y-axis is the inhibition z-score, while the right y-axis e is − log10 of p-value. The cutoff line for p-value is < 0.05.

RNA-seq analysis 14 days after injury with and without treatment (Fig. 1b) revealed well-clustered transcriptional profiles in each treated group (Fig. 2a). In principal component analysis (PCA) plots (Fig. 2a), the transcriptomic profile of non-injured corneas, control (red), and injured corneas treated with vehicle (green) were separated well. Topical treatment with lipid mediators shows profiles closer to control corneas than to vehicle-treated corneas. ELV-N32 (pink) was the nearest to the normal cornea, follow by RvD6i, LXA4, and NPD1. DEseq2 analysis allows comparison of all treated groups as well as control corneas to vehicle as a reference. Upregulated genes in vehicle-treated injury corneas revealed differences among treatment with lipid mediators, as depicted in Venn diagrams (Fig. 2b). Since NPD1 failed to decrease the ACE2 expression and RBD binding upon injury (Fig. 1d–h), we focused on the groups of shared genes (red circles) between control-LXA4-ELV-N32-RvD6i (450 genes including *Ace2*) and control-ELV-N32-RvD6i (737 genes). The EnrichR generating-KEGG pathway analysis of these two data sets revealed cytokines and senescence-related pathways (Fig. 2c) with significant false discover rate (FDR) values. On the other hand, IPA analysis of these two gene set combinations (1187 genes in total in Fig. 2b) predicted several cytokines as upstream regulators of increased *Ace2* expression after injury (Fig. 2d). In addition to cytokines, the senescence maker CDKN2A (p16/INK4) and the NFkB (complex) and its correlated genes were predicted to be inducers of *Ace2* (Fig. 2d). The RNA-seq analysis of the *Cdkn2a* gene (Fig. 2e), the IPA inhibition score and p-value of this gene (Fig. 2f), and the NFKB complex (Fig. 2g) confirm that ELV-N32 and RvD6i are the most potent inhibitors of these pathways.

### Lipid mediators counter-regulate pro-inflammatory cytokines, NFkB/inflammation, and senescence-associated secretory phenotype after cornea injury

**Figure 3:**
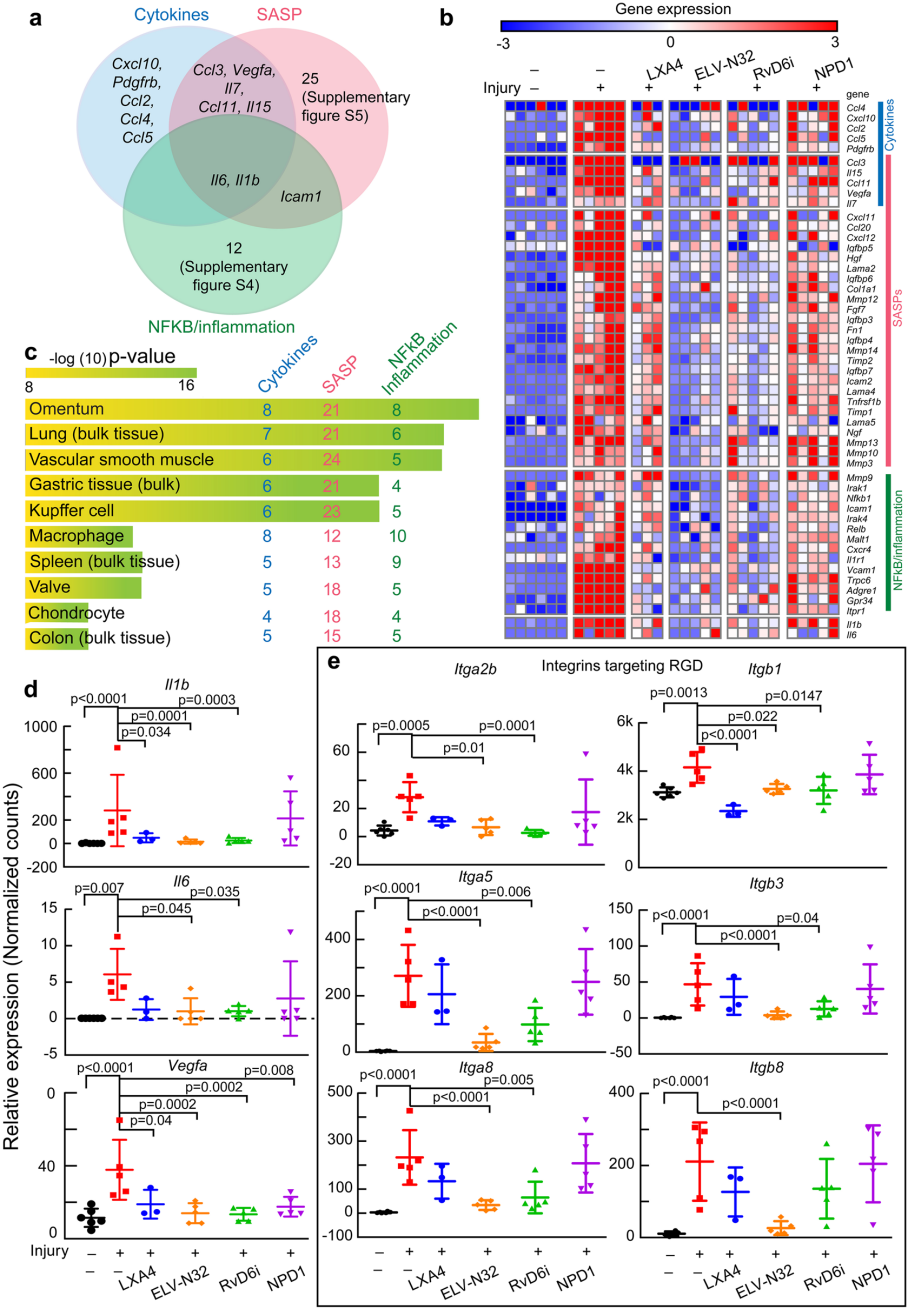
Lipid mediators down-regulate injury-induced gene expression of NFkB/inflammation, senescence-associated secretory phenotype, and pro-inflammatory cytokines after cornea injury. (**a**) Venn diagram of cytokines, SASP, and NFkB inflammatory genes upregulated by injury. (**b**) Heatmap of normalized counts data. Each small square represents data from one cornea. There are 51 genes increased by injury, and most are inhibited by ELV-N32 and RvD6i treatment. (**c**) The ArchS4 human tissue analysis prediction for the 51 genes. The length of the bar represents the significance of the gene set in the tissues, while the lighter the color, the higher the significance. The numbers show the genes from the denoted group enriched in each pathway. (**d**) Scatter plots of *Il1b*, *Il6*, and *Vegfa* genes. (**e**) Scatter plots of genes that encode proteins that target RGD. The p-value of ANOVA-post hoc Dunnett's multiple comparisons test with vehicle as reference are shown. Mean and SD are depicted as the lines. The normalized counts were used for analysis. The protein level of IL1B and VEGF were confirmed using Jess capillary-based western assay (Supplementary Fig. S8).

Since upregulated *Ace2* gene expression is caused by the action of cytokines, p16INK4a, and NFkB, we targeted genes regulated by these inducers. Thus, in the injured cornea, we explored: (i) activated cytokines found in the serum of SARS-CoV-2 patients^22^, (ii) senescence-associated secretory phenotype (SASP) genes^25^, and (iii) NFkB/inflammation genes found in lung biopsies of SARS-CoV-2^26^. The Venn diagram showed several genes shared by the three inducers (Fig. 3a). The Heatmap indicated that 51 injury-upregulated genes were counteracted by the lipid mediators (Fig. 3b). All 51 of these genes belong to the cytokines and SASP-related genes (Supplementary Fig. S3a). The plot for each specific gene is provided in Supplementary Figs. S3–S5. Among those genes, *Cxcl10*, *Il1r1*, and *Hgf* (Supplementary Figs. S3c, S4, and S5) are related to SARS-CoV-2 severity^27^, while genes related to metalloproteinases, such as *Mmp9* (Supplementary Fig. S4), *Mmp3*, *Mmp12*, and *Timp1* (Supplementary Fig. S5), are increased after coronavirus infection and involved in the degradation of the extracellular matrix, which facilitates hyper-inflammation, leukocyte infiltration, and ECM remodeling and fibrosis^28,29^. Further, transient receptor *Trpc6* (Supplementary Fig. S4) is a component of chronic obstructive pulmonary disease development^30^.

Using pathway analysis, we found similar pathways to those found in the entire transcriptome (Fig. 2c and Supplementary Fig. S3a). Employing the EnrichR–Archs4 human analysis tissue database, we found that the 51 genes are more abundant in the omentum and lung (bulk tissue) (Fig. 3c). This suggests that genes detected in the injured cornea might recapitulate changes in gene expression that occur after lung injury. Three targeted cytokines, *Il1b*, *Il6*, and *Vegfa* genes, are plotted in Fig. 3d. Our data showed that these genes were upregulated by the injury, and the administration of LXA4, ELV-N32, or RvD6i reduced their expression. We also focused on integrin genes since the Spike protein contains an arginine-glycine-aspartic acid motif in the RBD site that is recognized by some integrins as a potential receptor of SARS-CoV-2^31,32^. Six integrins, which have the RGD binding domain in the heterodimer conformation, are increased after injury and decreased by some of the lipid mediators (Fig. 3e). Among these genes, *Itga5* and *Itgb1* are of interest since their specific blocker ATN-161 greatly attenuates the SARS-CoV-2 infection in vitro^33^, and their expression is significantly decreased by ELV-N32 and RvD6i.

### Lipid mediators attenuate IFNγ-specific induction of ACE2 expression, RBD exposure, and senescence programming in HCEC

**Figure 4:**
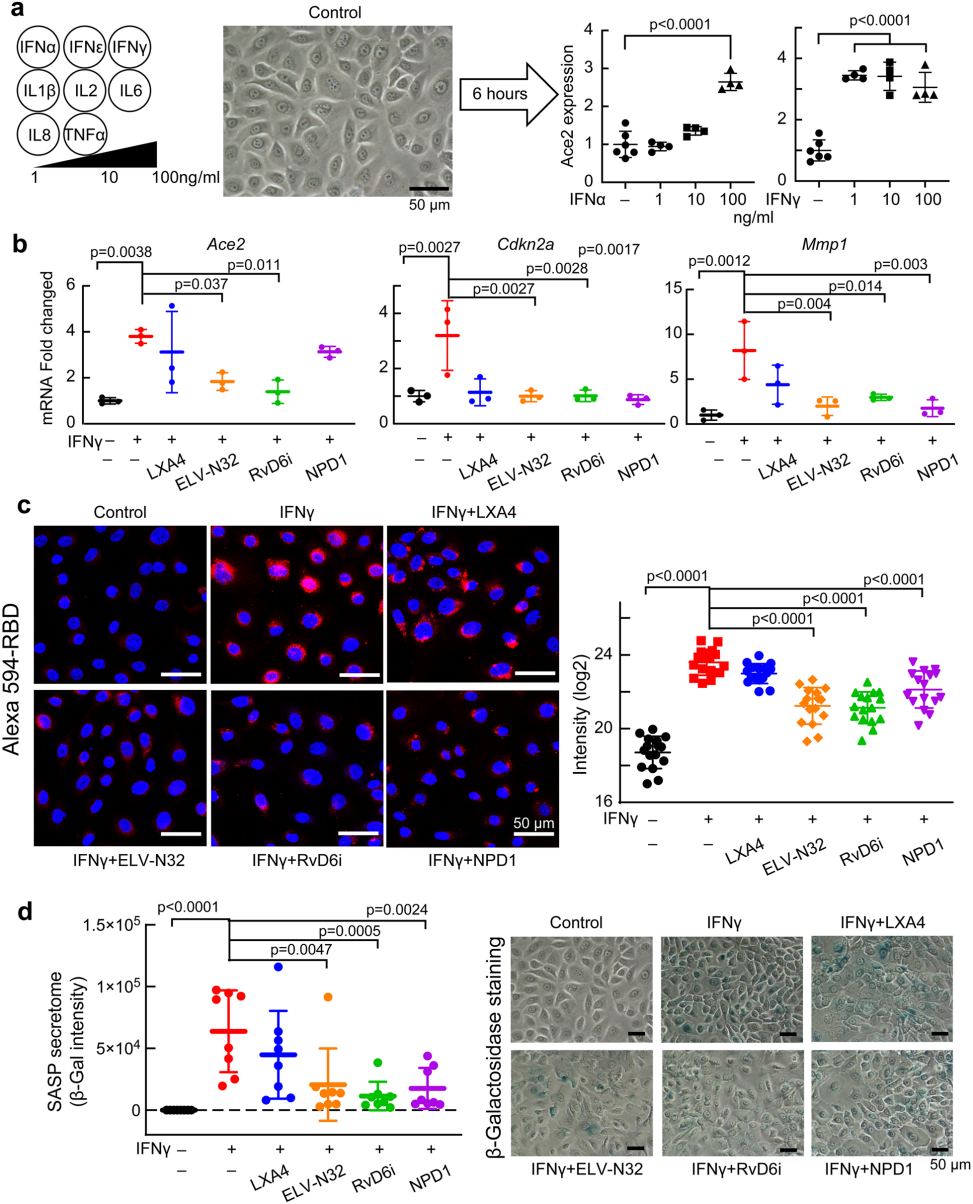
Lipid mediators attenuate IFNγ-induced ACE2 expression, senescence programming, and binding of Alexa 594-RBD in human corneal epithelial cells (HCEC). (**a**) Among several cytokines tested only IFNγ and α, induces ACE2 expression in HCEC (6 h after stimulation, analyzed by dd-PCR). (**b**) Effect of lipid mediators on gene expression of *Ace2*, *Cdkn2a*, and *Mmp1* of HCEC after adding IFNγ (100 ng/mL). ΔΔC_T_ normalized fold change was used. p-values of statistical *t* test analysis in comparison to vehicle group are shown. Mean and SD are shown as the lines. (**c**) Alexa 594-RBD binding in HCEC. IFNγ (100 ng/mL) and lipid mediators (200 nM) were added to the HCEC for 12 h. Alexa 594-RBD (0.5 ng/well) was then added and images taken 24 h after. Fifteen images/condition analyzed. Representative images are shown (left side), and the Imaris based calculation was plotted (right-hand side). Data are presented as single image/each data point. The p-value of ANOVA-post hoc Dunnett's multiple comparisons test with vehicle as reference. Mean and SD are shown as the lines. (**d**) SASP Secretome (β-Gal staining) of HCEC 24 h after IFN-γ challenge and ± lipid mediators. Each point represents one image. The p-value of ANOVA-post hoc Dunnett's multiple comparisons test with vehicle as reference are shown. Mean and SD are shown as the lines. Representative images for each condition are in the right panel.

Based on the IPA prediction that upstream regulators of *Ace2* targeted some cytokines, we treated HCEC with IL1β, IL2, IL6, IL8, IFNγ, IFNα, IFNε, or TNFα at 1, 10, and 100 ng/mL concentrations. IFNγ and IFNα were the only cytokines to activate *Ace2* expression, with IFNγ being the more potent of the two, inducing ACE2 expression at 1 ng/mL. Therefore, we chose IFNγ as the inducer of *Ace2* in the HCEC experiment (Fig. 4a and Supplementary Fig. S6). Following *Ace2* expression by dd-PCR that provides absolute quantification at the transcription level, ELV-N32 and RvD6i markedly attenuated IFNγ-triggered *Ace2* activation while LXA4 did not have any effect. (Fig. 4b). In addition, IFNγ stimulates the overexpression of senescence programming genes *Cdkn2a* (p16INK4a) and *Mmp1*. ELV-N32, RvD6i, and NPD1 decrease *Cdkn2a* activation to control values, but LXA4 does not. IFNγ-stimulated Alexa 594-RBD binding was analyzed by Imaris (Fig. 4c and Supplementary Fig. S7) correlates with increased ACE2 expression (Fig. 4b). ELV-N32, RvD6, and NPD1 decrease IFNγ-stimulated RBD binding (Fig. 4c). Following IFNγ-stimulated RBD binding to ACE2, induction of senescence programming genes *Cdkn2a* (p16INK4a) and *Mmp1*, and SASP secretome activation (β-Gal staining) takes place. These events are blocked by ELV-N32, RvD6i, and NPD1 but not by LXA4 (Fig. 4d).

## Discussion

The severe COVID-19 outbreak is characterized by hyper-inflammation and “cytokine storm,” therefore, the management of local and systemic inflammatory response to SARS-CoV-2 may be as important as antiviral therapies. Among anti-inflammatory agents, the specialized pro-resolvin lipid mediators (SPMs), such as LXA4, NPD1 and RvD6i, facilitate the clearance of cell debris and counteract the action of pro-inflammatory cytokines in a process called “inflammation resolution without any immunosuppressive complications”^34^. Recently, SPMs were hypothesized as potential COVID-19 treatments^9–11^. However, practical evidence, as well as working mechanisms, were not available. Here, we discern bioactivity among a group of lipid mediators on ACE2 expression, pro-inflammatory cytokines, and senescent proteins, which are critical targets related to SARS-CoV-2 entrance and deleterious consequences of this viral infection. We uncover that the lipid mediators ELV-N32 and RvD6i decrease ACE2 receptor expression and binding of RBD of the S protein, preventing enhancement of cytokine expression and senescence programming using a rat cornea in vivo model of alkali burn that induces a strong inflammatory reaction. Using HCEC in culture challenged with IFNγ, we found that ELV-N32 and RvD6i exert similar effects. In addition, ELV-N32 remarkably decreases Furin expression, a protease that cleaves the S1/S2 site required for SARS-CoV-2 entry in lung cells^6^.

A key cytokine responding to viral infections is IFNγ^35^, which increases in the serum of severely affected COVID-19 patients^22,36^. We found that IFNγ induces *Ace2* expression in HCEC at a much lower dose than IFNα and increases the binding of the RBD to the cells. Moreover, IFNγ activates cellular senescence reflected in enhanced *Cdkn2a* expression and SASP secretome release. This observation could contribute to explaining why aging populations are more susceptible to COVID-19^37^. ELV-N32 does bear senolytic activity^19^, and both ELV-N32 and RvD6i suppressed senescence genes and SASP secretome in HCEC (Fig. 4d). Therefore, S protein internalization may lead to IFNγ secretion, which would synergize with an integrin-rich environment to amplify the IFNγ effect^38^ and stimulate *Ace2* overexpression. As a result, the higher the ACE2, the higher the possibility of SARS-CoV-2 binding will be. ELV-N32 and RvD6i suppressed the IFNγ stimulation of Ace2 expression as well as the IFNγ-induced senescence, where many SASP components are pro-inflammatory cytokines. PEDF + DHA (the precursor of RvD6i) and RvD1 suppress type 1 pro-inflammatory macrophages (induced by IFNγ) while increasing the type 2 anti-inflammatory macrophage phenotype^39,40^. Interestingly, ELV-N32, RvD6i, and NPD1 attenuated the binding of ACE2-RBD in the IFNγ-treated cells in culture (Fig. 4c), while in the rat injured cornea, LXA4 displayed a significant effect on preventing ACE2-RBD interaction (Fig. 1f–h). Of the lipid mediators studied, ELV-N32 and RvD6i are the two most consistent lipid mediators displaying protective bioactivity. RvD6i was recently identified in mouse tears as related to corneal nerve regeneration^17,41,42^. ELV-N32 is a powerful neuroprotective and anti-inflammatory lipid mediator^18^.

Tissue damage is a serious complication of SARS-CoV-2-infected patients. Recently, Rosa et al*.* demonstrated that, in both tuberculosis and COVID-19 (two pulmonary diseases where neutrophils are associated with increased severity), there is an upregulation of neutrophil degranulation, innate immune response, ACE2 level, and IFNγ signaling pathways^43^. These results are similar to what we found in our cornea alkali-burn-damage model. Therefore, corneal alkali burn as a tissue damage model mimics the physical response of intestinal or lung epithelial cells during and after SARS-CoV-2 infection.

ACE2 is an anti-inflammatory factor^44^ and is also a SARS-CoV-2 receptor^4^. Thus, the increased abundance of ACE2 in damaged tissue, such as the lung or the cornea in this study, is a double-edged sword, especially when the anti-inflammatory activity is inadequate in diminishing the pro-inflammatory cytokines produced by the infection. The lipid mediators ELV-N32 and RvD6i might be an alternative way of providing anti-inflammatory activity. Even though the action of LXA4 or NPD1 was not consistent between in vitro and in vivo models, the potential of using lipid mediators to maintain the homeostasis after tissue damage is remarkable, especially in the current scenario in which the ‘long COVID’ (the long-lasting health effects of SARS-CoV-2 infection) is not well-documented.

ELV-N32 and RvD6i also decrease integrin expression. The S protein contains an RGD motif in the RBD site that recognizes integrins and stimulates virus internalization by activation PI-3K^31,32^, a pathway that the KEGG analysis from EnrichR tool predicted to increase along with ACE2 enhanced expression (Fig. 2c). The inhibition of integrin α5β1 by a non-RGD peptide derived from fibronectin prevents the binding of the S protein to ACE2 and decreases virus infection in vitro^33^.

Recently, Miner et al*.* showed that the human cornea does not support SARS-CoV-2 infection despite the expression of ACE2 in the human corneal epithelium^45^. However, studies in COVID-19 patients found an increase of the virus in dry eye disease, suggesting a possible consequence of infection^46^, and in a cohort of patients with COVID-19, 63% had a positive viral test in the conjunctiva, but the origin of the infection was undetermined^47^. Nevertheless, the role of lipid mediators in managing the ACE2 upregulation as well as hyper-inflammation, increase in cytokine expression, and senescence programming after tissue damage uncovers mechanistic insights that are also applicable to other tissues.

In conclusion, our data demonstrate that ELV-N32 or RvD6i diminish ACE2 expression and binding of the S protein RBD and, consequently, activate pro-homeostatic signaling and reduce tissue damage.

The application of these lipid mediators alone or as a complement with current antiviral strategies for COVID-19 could be of therapeutic use. Moreover, the lipid mediators identified here might work by similar mechanisms in other cell types and further expand the scope of their therapeutic applications.

### Limitations of this study

Additional research will be needed to fully elucidate the molecular mechanisms by which the lipid mediators downregulate ACE2 and the genes encoding inflammatory/senescence proteins. The use of the full-length S protein or the pseudotype-virus that expresses SARS-CoV-2 S protein in the models studied here will provide insights into the molecular connection between lipid mediators acting on the cell attachment and cell entrance of the SARS-CoV-2 virus, particularly since ELV-N32 remarkably reduces furin expression correlated with ACE2 downregulation. Moreover, the use of the intact virus would offer a direct demonstration of the significance of the effects of the lipid mediators studied here.

## Methods

### Animals

Sprague–Dawley rats (8-week-old male) were obtained from Charles River Laboratories (Wilmington, MA, USA) and kept at the Animal Care of the Neuroscience Center of Excellence, Louisiana State University Health (LSUH; New Orleans, LA, USA). All animals were handled in compliance with the guidelines of the ARVO Statement for the Use of Animals in Ophthalmic and Vision Research, and ARRIVE guidelines (https://arriveguidelines.org). The experimental protocol was approved by the Institutional Animal Care and Use Committee (IACUC) at LSUH.

### Cornea injury

The rats were anesthetized by intraperitoneal injection of Ketamine (50–100 mg/kg) plus xylazine (5–10 mg/kg). A 4 mm diameter filter paper soaked in 1 N NaOH was placed on the central cornea of the right eye for 45 s, and then the eye was thoroughly washed with 10 mL of saline. After injury, the rats were randomly divided into five treatment groups: vehicle; lipoxin A4 (LXA4) from Cayman Chemical (Ann Arbor, MI, USA); R,R Resolvin D6 isomer (RvD6i), R,R neuroprotection D (NPD1), and elovanoid (ELV)-N32 synthesized by Dr. R. Nshimiyimana, T.F. Lam, and Prof. N. Petasis. All lipid solutions were prepared daily at the final concentration of 10 μM using PBS with the minimal contamination of ethanol by evaporating the ethanol and immediately dissolve the lipids in PBS, then vortex well for 2 min. Topical administration (20 μL) was done 3 ×/day for 14 days. To determine if the lipid have any side effects in the non-injured rat eye, eye drops at the same concentrations were used 3 ×/day for 1 week. The treatment did not produce blinking or rubbing of the eyes. There were no signs of conjunctiva hyperemia or edema using slit-lamp examination. The experiments were double-blinded with the lipid mediators coded during the whole experiments. At the end of the study, when all data was collected, the code was opened.

### Antibodies

See Table 1.

### Corneal RNA-sequencing

Injured corneas (n = 5/condition) were harvested and homogenized with TRIzol (ThermoFisher Scientific) on ice with a glass Dounce homogenizer. RNA sequencing was performed as described^17^. Briefly, after mRNA extraction and determination of purity, 8 ng of total RNA was reverse transcribed, and total cDNAs were amplified using ISPCR primer, and the library was made with the Nextera XT DNA library preparation kit (Illumina, San Diego, CA, USA). The libraries were pooled with the same molarity and sequenced using the NextSeq 500/550 High Output Kit v2 (75 cycles, Illumina). After demultiplexing, RNA-seq data were aligned to the Rattus Norvegicus reference genome (ftp://ftp.ensembl.org/pub/release-98/fasta/rattus_norvegicus/dna/) using the Subread package v2.0.1 alignment function^48^. The BAM files for sequencing data alignment were counted using featureCounts function of Subread tool^49^ using the macOS Catalina. The raw count data were subjected to differential gene expression analysis using DESeq2 package for R^50^ with the vehicle group as reference. The adjusted p-values were named as the false discover rate (FDR). Significantly changed genes (FDR < 0.05) between each treatment vs. vehicle were subjected to the enrichment analysis using EnrichR^51^ and NetworkAnalyst 3.0^52^, and pathway analysis using the IPA (Qiagen Inc., https://www.qiagenbioinformatics.com/products/ingenuity-pathway-analysis).

### Preparation of Alexa 594-conjugated RBD fragment of S protein

RBD fragment of the Spike protein belonging to SARS-CoV-2 (Raybiotech, Peachtree Corners GA. Cat. 230-30162-1000) was labeled using Alexa Fluor 594 Protein Labeling Kit (ThermoFisher Scientific, Waltham, MA. Cat. A10239) following the manufacturer’s directions. Briefly, 1 mg of protein was dissolved in 0.1 M bicarbonate and then incubated with the Alexa Fluor 594 dye for one hour. The dye was washed using an Amicon-Ultra centrifugal filter cutoff 10 kDa (Merck, Millipore Carrigtwohill, CO. Cat. UFC201024). To assess the efficiency of the label, the protein was measured at 280 nm and 590 nm absorbance using NanoDrop One (ThermoFisher Scientific). There was a ratio of 0.4 mol of dye/mole of protein and a recovery of about 80%.

### Human corneal epithelial cells (HCEC) culture

All experiments with human corneal epithelial cells were approved by the Institutional Review Board of LSUHNO and conducted in accordance with NIH guidelines. The HCEC were obtained by Dr. Roger Beuerman using an HPV16-E6E7 vector and were kept frozen in the laboratory at passage 25^53^. Cells were maintained in keratinocyte growth (KGM) medium containing the keratinocyte basal medium (KBM) (Lonza: CC-3101) supplemented with bovine pituitary extract (BPE), hEGF, Insulin, Hydrocortisone and Gentamicin Sulfate-Amphotericin (GA-1000) (Lonza, Cat. CC-4131). For all experiments, cells were seeded at 30,000 cells/cm^2^.

For screening the stimulation of receptor ACE2 by cytokines, the HCEC were cultured with KGM until 50–60% confluence. Then, changed to KBM containing IL-1β, -2, -6 and 8, IFN-α, -ε, and -γ or TNFα at 1, 10 or 100 ng/mL. The cells were harvested after 6 h and analyze for the gene expression of *Ace2*. In other experiments, HCEC were stimulated with IFNγ, and thereafter, lipid mediators were added. For the Alexa 594-conjugated RBD binding, IFNγ was used as a cytokine trigger. At 12 h after cytokine exposure and lipid mediator treatments, 0.5 μg of labeled RBD was added to the medium. The evaluation of RBD binding was conducted 24 h after.

### Immunohistochemistry

Corneal tissue was fixed in Zamboni fixative (MasterTech Scientific, Lodi, CA USA) for 2 h immediately after euthanasia. After thoroughly washing with PBS, the corneas were embedded in optimal cutting temperature compound, and serial 10-μm cryostat sections were obtained, dried at room temperature for 2 h, and stored at − 20 °C until use. For immunofluorescence, the sections were incubated with primary antibodies at the concentration described in Table 1 in a wet chamber at 4 °C overnight. The sections were washed 3 ×/5 min with PBS following by incubation for 1 h at RT with Alexa Fluor-conjugated secondary antibodies (1:1000 dilution). All sections were counterstained with DAPI (ThermoFisher Scientific, Cat. D1306), and images of rat corneal samples were acquired with an Olympus IX71 fluorescent microscope.

**Table 1:** List of primary antibodies used in this study.

No.	Name	Company	Cat. number	Immunofluorescence	Western blot
1	Rabbit anti-ACE2	Abcam	Ab108252	1:1000	1:100
2	Rabbit anti-DPP4	Abcam	Ab129060	1:500	1:100
3	Rabbit anti-FURIN	Abcam	Ab183495	1:1000	1:100
4	Rabbit anti-TMPRSS2	Abcam	Ab109131	1:1500	1:100
5	Rabbit anti-GAPDH	Santa Cruz Biotechnology	Sc-25778		1:1000
6	Anti-neutrophil	LSBio	LS-C348005	1:500	
7	Mouse anti-rat CD68	Bio-Rad	MCA341GA	1:1000	
8	Rabbit polyclonal to IL-1 beta	Abcam	Ab9722		1:100
9	Rabbit polyclonal to IL-6	Abcam	Ab6672		1:100
10	Rabbit polyclonal to MMP14	Abcam	Ab53712		1:100

### Unbiased imaging-based evaluation of RBD binding

Twenty-four hours after Alexa 594-RBD was added to the HCEC, the cells were washed with PBS (3 ×/5 min) and fixed with 4% paraformaldehyde for 30 min at RT. The HCEC were washed 2 × with PBS and stained with Hoechst 33342 Solution (ThermoFisher Scientific, Cat. 62249) for 30 min at RT. Next, the HCEC were washed 2 × with PBS before imaging. For unbiased data collection, 7 designated areas were defined in each well (Supplementary Fig. S7) and captured with an Olympus FV3000 confocal laser scanning microscopy under “Multi Area Time Lapse” (MATL) mode. All images were acquired with the same parameters and Z-section range, converted and inputted in the Imaris software version 9.5.1. The threshold for the control images was defined by the HCEC without Alexa 594-conjugated RBD of S protein and using it as a threshold filter for the Imaris batch image processing function. The sum of total intensity for each image was used to evaluate the binding efficiency. The whole process was summarized in Supplementary Fig. S7b,c.

### Droplet digital PCR (dd-PCR)

Total RNA was isolated using RNeasy Plus Mini Kit (Qiagen, Germany), and 1 µg of total RNA was reverse transcribed using an iScript cDNA Synthesis Kit (Bio-Rad, Cat. 170–8841). For ddPCR, 10 ng of cDNA was multiplexed with *Ace2* and *phosphoglycerate kinase 1* (*Pgk1*) probes (Bio-Rad, Cat. qHSACEP005-1563 and dHSACPE503-3809) using dd-PCR Supermix for Probes No dUTP (Bio-Rad, Cat. 1863024). Then, 20 µL of the reaction was mixed with 70 µL of Droplet Generation Oil (Bio-Rad Cat. 1863005) to make the reaction droplets. The emulsified samples were carefully transferred to PCR plates (Bio-Rad, Cat. 12001925) and amplified using the cycling: 95 °C for 10 min, 40 cycles of a two-step cycling protocol (94 °C for 30 s and 60 °C for 1 min), and 98 °C for 10 min. Next, the post-cycling plate was placed into the QX200 Droplet Reader with the FAM/HEX setting. The absolute quantity of DNA per sample (copies/µL) was processed using QuantaSoft Analysis Pro Software. For the data analysis, the ratio of quantified *Ace2* to *Pgk1* was used.

### Capillary-based western blot

The capillary-based western assay was performed using a Jess system (Protein Simple, San Jose, CA, USA) as manufacture suggested protocol. Briefly, samples were lysed with RIPA buffer containing a protease inhibitor cocktail (Sigma, Cat. P8340). Cell debris was removed after 10 min centrifugation at 16,000×*g*. Protein concentration was determined by BCA assay (ThermoFisher Scientific, Cat. 23225) and 1 µg was used/reaction. Fluorescent Master Mix was mixed with 40 mM DTT, and the mixture was added to each sample (1 µg/5 µL) to provide a denaturing and reducing environment. Samples were heated at 95 °C/5 min, and 3 µL of each sample (0.6 µg of total protein) were loaded. The 12–230 kDa cartridge (Protein Simple, #SM-W004) was used. Primary antibodies were diluted in antibody diluent 2 buffer (Protein Simple, #042-203) while the working solution of secondary antibodies was provided by the company (Protein Simple, #042-206). Then, the filled plate was spin-down for 10 min at 1000×*g* to remove bubbles and plate, and capillaries were loaded into the Jess machine. For data analysis, the area of spectra that matched the molecular weight of the target protein was used. To reduce the coefficient variant, we analyzed the GAPDH for each capillary. The ratio of the targeted protein to GAPDH was used for statistical comparisons. For visualization, the artificial lanes generated from spectra volume was used.

### High-throughput qPCR using Biomark HD

Quantitative PCR was performed with the Biomark HD system (Fluidigm, San Francisco, CA, USA). Briefly, 200 ng of RNA was reverse-transcribed using iScript Reverse Transcription Supermix (Bio-Rad), and the cDNA was pre-amplified using the PreAmp Master Mix (PN 100-5580; Fluidigm). The cDNA was then subjected to Exonuclease I treatment and diluted 5 times in TE Buffer. The qPCR reaction mixture and primer reaction mixture were made and loaded into the Biomark 96.96 IFC (Integrated Fluidic Circuit). The enzyme reaction was mixed using Juno Controller (Fluidigm) and run using the cycling program of (i) 70 °C for 40 min followed by 60 °C for 30 s, (ii) hot start for 1 min at 95 °C, (iii) 30 cycles of denaturation at 96 °C for 5 s, and annealing at 60 °C for 20 s, and (iv) melting curves between 60 °C and 95 °C with 1 °C increments/3 s. The Ct value of target genes was normalized to the house-keeping genes *Gapdh*, *Hprt1*, and *Tfrc* before normalized to the vehicle group. Relative fold changes from the ΔΔC_T_ calculation was used to make the graph. Primer sequences are provided in Table 2.

**Table 2:** Primers for qPCR.

Gene name	Forward	Reverse
*Ace2*	CATTGGAGCAAGTGTTGGATCTT	GAGCTAATGCATGCCATTCTCA
*Cdkn2a*	GGGGGCACCAGAGGCAGT	GGTTGTGGCGGGGGCAGTT
*Mmp1*	GGGCTTGAAGCTGCTTACGAATT	CAGCATCGATATGCTTCACAGTTCT
*Gapdh*	TGGACCTGACCTGCCGTCTA	CCCTGTTGCTGTAGCCAAATTC
*Tfrc*	GGCTACTTGGGCTATTGTAAAGG	CAGTTTCTCCGACAACTTTCTCT
*Hprt1*	GACCAGTCAACAGGGGACAT	AACACTTCGTGGGGTCCTTTTC

### Statistical analysis

Data are expressed as mean ± SD. The data were analyzed by 1-way ANOVA followed by Dunnett's multiple comparisons post hoc test at 95% confidence level with the vehicle as reference. All graphs were made using GraphPad Prism 7 (GraphPad Software, La Jolla, CA, USA) with the mean ± SD, while all statistical analyses were done using the built-in function of Prism 7.

## Supplementary Information

Supplementary Figures.

## Data Availability

The data that support the findings of this study are available from the corresponding authors upon reasonable request.
