# Supplementary material for: ELV-N32 and RvD6 isomer decrease pro-inflammatory cytokines, senescence programming, ACE2 and SARS-CoV-2-spike protein RBD binding in injured cornea
Source: Sci Rep. 2021 Jun 17;11:12787. doi: 10.1038/s41598-021-92293-x (PMC8211643; doi:10.1038/s41598-021-92293-x)
Supplement: Supplementary file 1 — Supplementary Figures. [file 41598_2021_92293_MOESM1_ESM.docx]

**ELV-N32 and RvD6 isomer decrease pro-inflammatory cytokines, senescence programming, ACE2 and SARS-CoV-2-spike protein RBD binding in injured cornea**

Thang L. Pham^1^, Jiucheng He^1^, Azucena H. Kakazu^1^, Jorgelina Calandria^1^, Khanh V. Do^1^, Robert Nshimiyimana^2^, Ting F. Lam^2^, Nicos A. Petasis^2^, Haydee E.P. Bazan^1^* and Nicolas G. Bazan^1^*

^1^Neuroscience Center of Excellence, School of Medicine, Louisiana State University Health New Orleans, New Orleans, LA, USA.

^2^Department of Chemistry and Loker Hydrocarbon Research Institute, University of Southern California, Los Angeles, CA, USA.

*Correspondence to: hbazan1@lsuhsc.edu and/or nbazan@lsuhsc.edu.


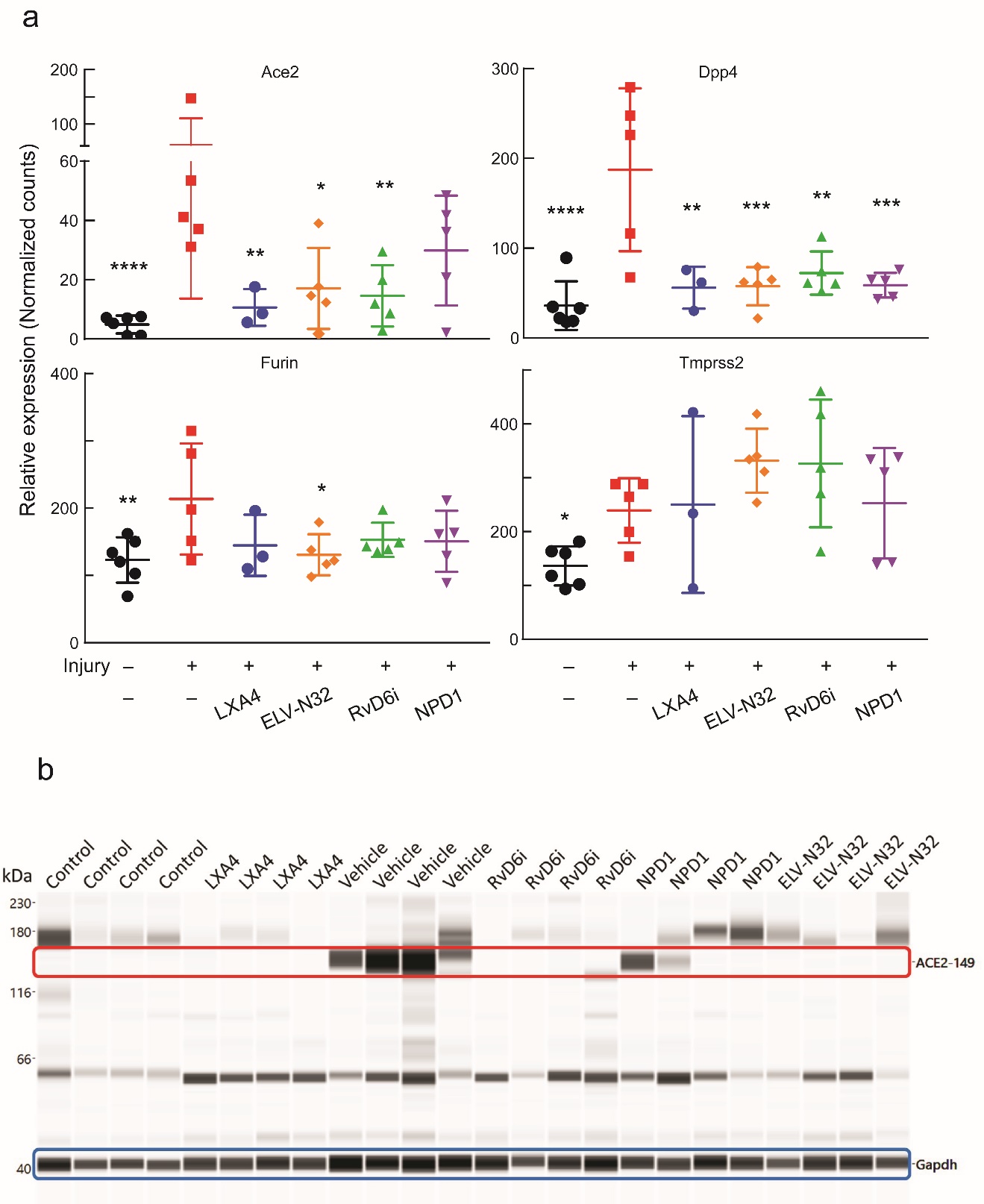
**Supplementary Figures**

**Supplementary Fig. S1.** **a**, Effect of lipid mediators on gene expression of *Ace2*, *Dpp4*, *Furin*, and *Tmprss2* after cornea injury. RNA-seq data normalized counts, (mean and SD) analyzed by ANOVA-post hoc Dunnett's multiple comparisons test with vehicle as reference. *, p < 0.05, **, p < 0.01, ***, p < 0.001, and ****, p < 0.0001. **b**, The whole blot of ACE2 protein. This image was cropped to make images presented in Figure 1**d**.


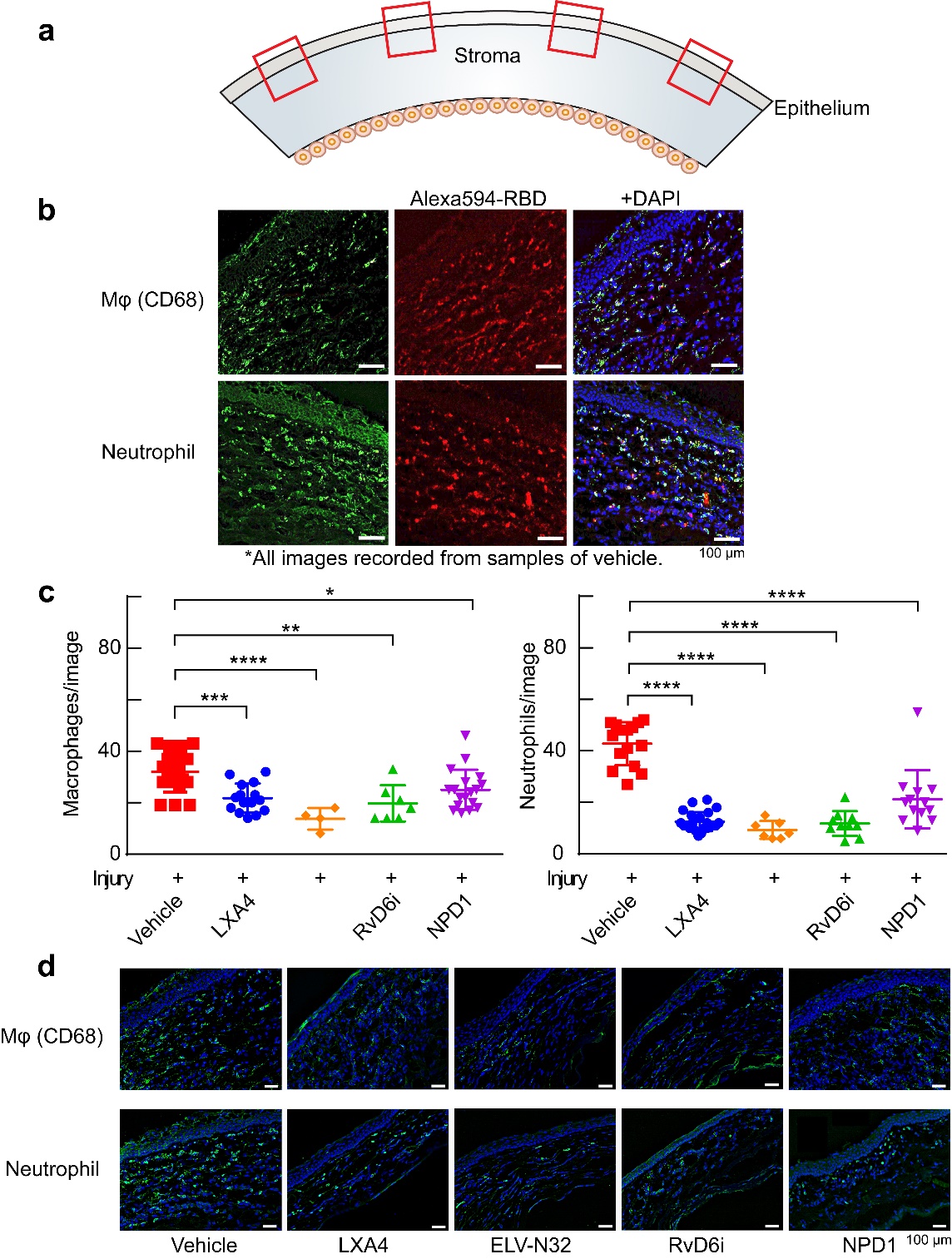


**Supplementary Fig. S2.** **a**, Illustration of unbiased microscopy analysis of the rat cornea. Four images were taken (red boxes) from cornea sections. **b**, Representative images stained with CD68 and Neutrophil antibodies and with Alexa 594-RBD of a rat injured cornea treated with vehicle. DAPI used to label the nuclei. **c,** Quantification of macrophage (+CD68 cells) and neutrophil. Each data point represents number of cells/cross-section image. Values are means ±SD and p-values calculated by ANOVA-post hoc Dunnett's multiple comparisons test with vehicle as reference. *, p < 0.05, **, p < 0.01, ***, p < 0.001, and ****, p < 0.0001. **d**, Representative immunofluorescence images of anti-CD68 macrophages and anti-neutrophils in treated groups. Green color for positive staining and blue for DAPI.


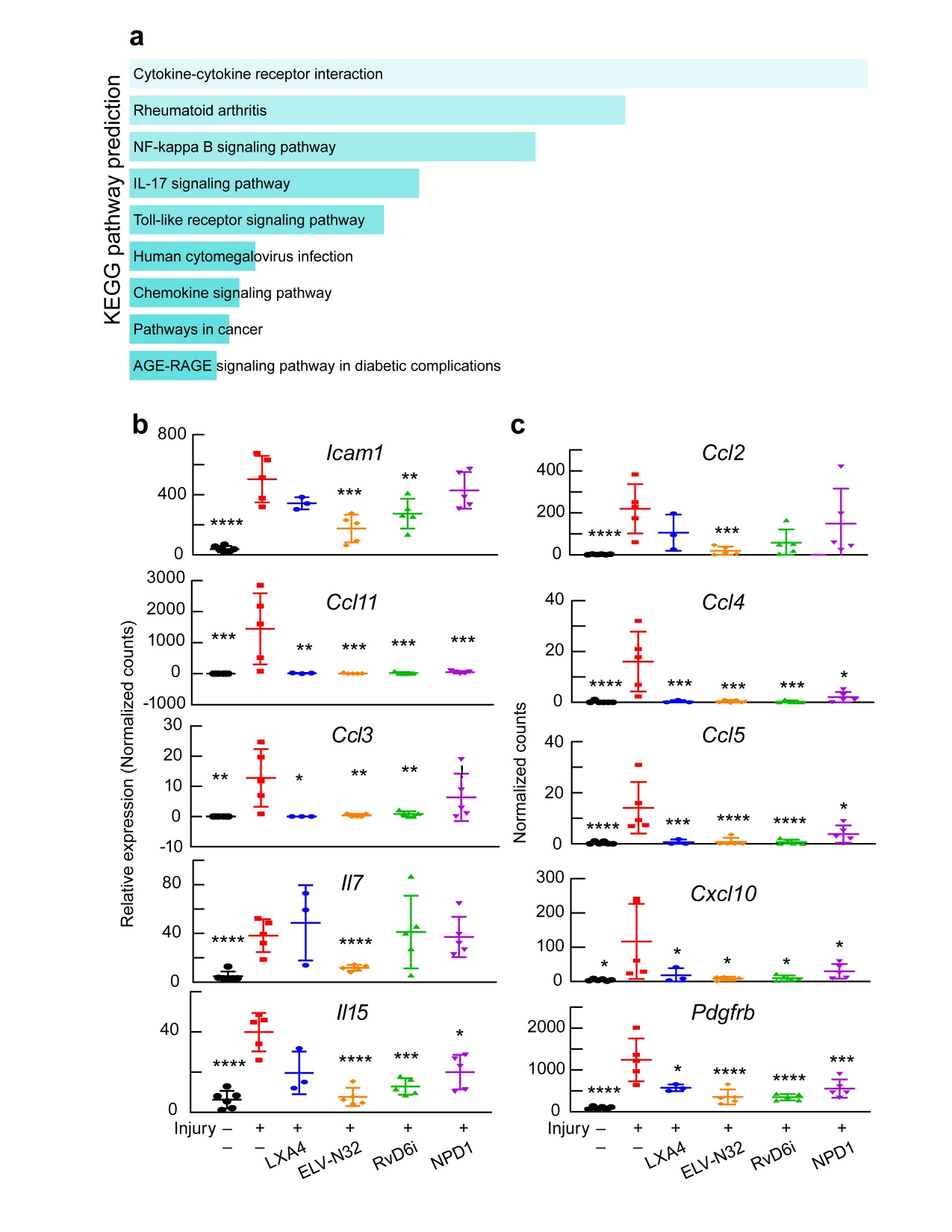


**Supplementary Fig. S3.** Lipid mediators attenuate expression of pro-inflammatory cytokines and SASP-related genes after cornea injury. **a**, KEGG-pathway enrichment of 51 genes depicted in Fig. 3**b**. Bars were sorted by p-value. The length of the bar represents the significance of the pathway, while the lighter the color, the more significant. **b**, RNA-seq gene expression share between pro-inflammatory cytokines and SASP. **c**, RNA-seq gene expression of pro-inflammatory cytokine genes. Normalized counts (mean and SD) analyzed by ANOVA-post hoc Dunnett's multiple comparisons test with vehicle as reference. *, p < 0.05, **, p < 0.01, ***, p < 0.001, and ****, p < 0.0001.


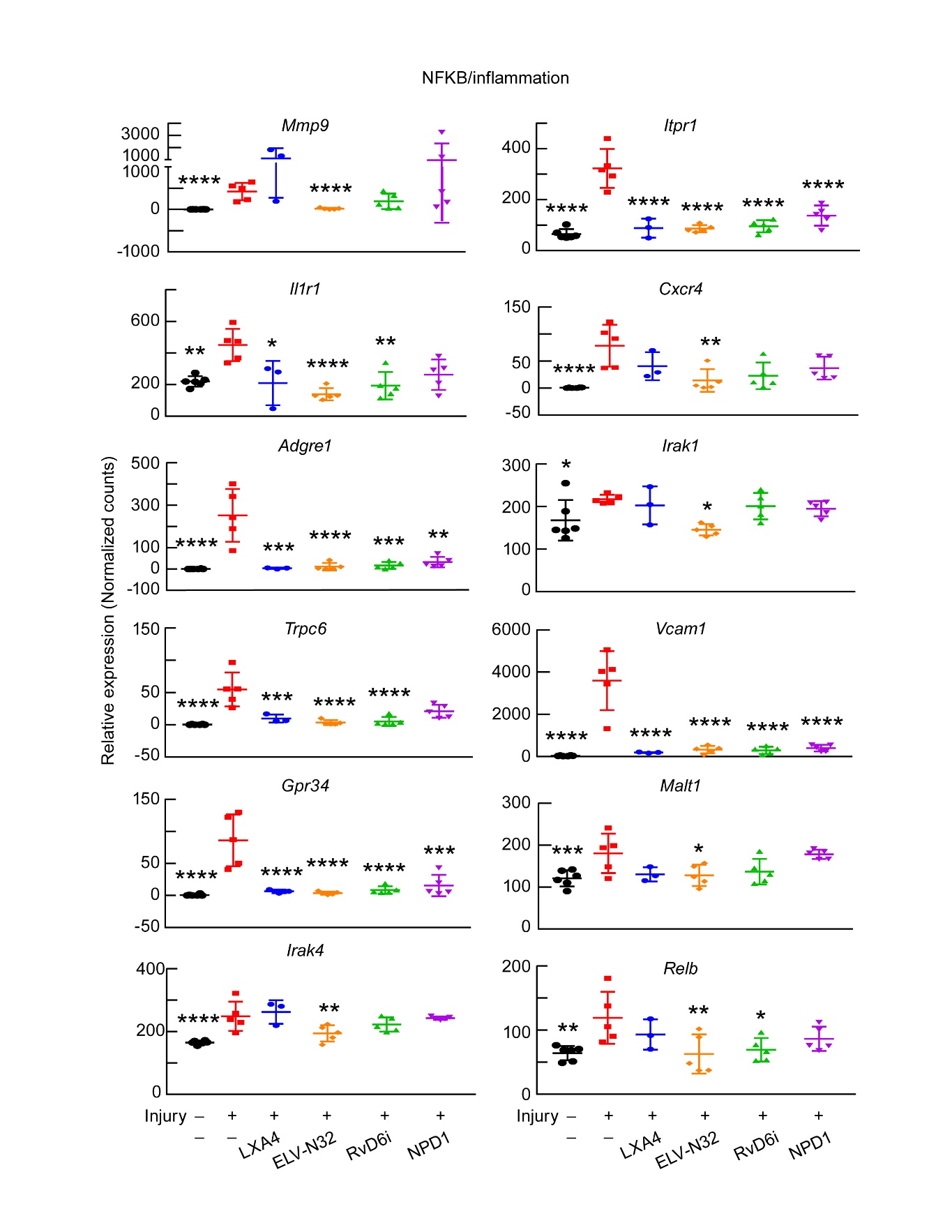
**Supplementary Fig. S4.** Effect of lipid mediators on expression of NFkB inflammatory genes after cornea injury. Normalized counts, (mean and SD) analyzed by ANOVA-post hoc Dunnett's multiple comparisons test with vehicle as reference *, p < 0.05, **, p < 0.01, ***, p < 0.001, and ****, p < 0.0001.


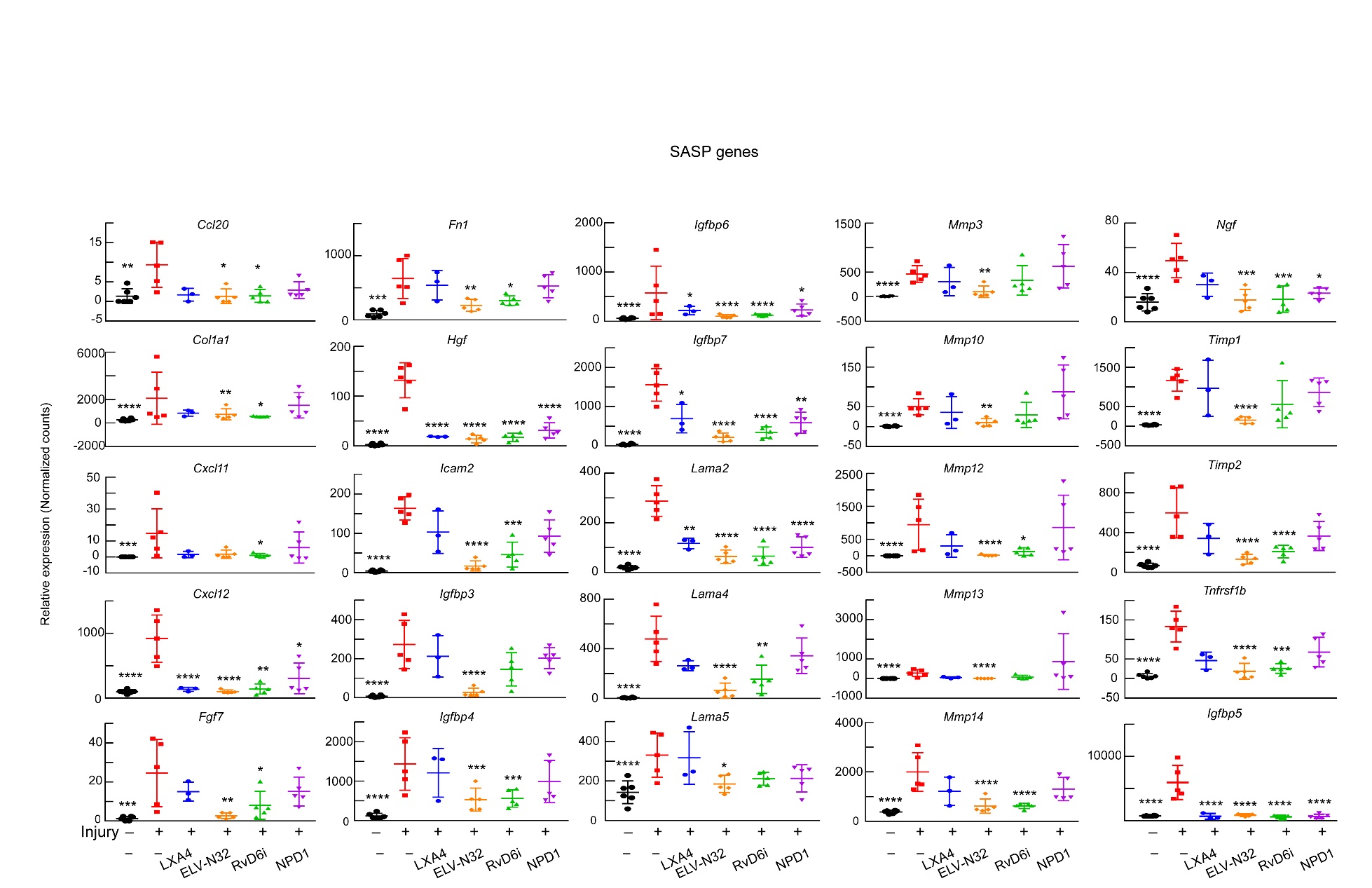


**Supplementary Fig. S5.** Differential effect of lipid meditators on senescence programming gene expression after cornea injury, RNA-seq gene expression. Normalized counts, (mean and SD) analyzed by ANOVA-post hoc Dunnett's multiple comparisons test with vehicle as reference. *, p < 0.05, **, p < 0.01, ***, p < 0.001, and ****, p < 0.0001.


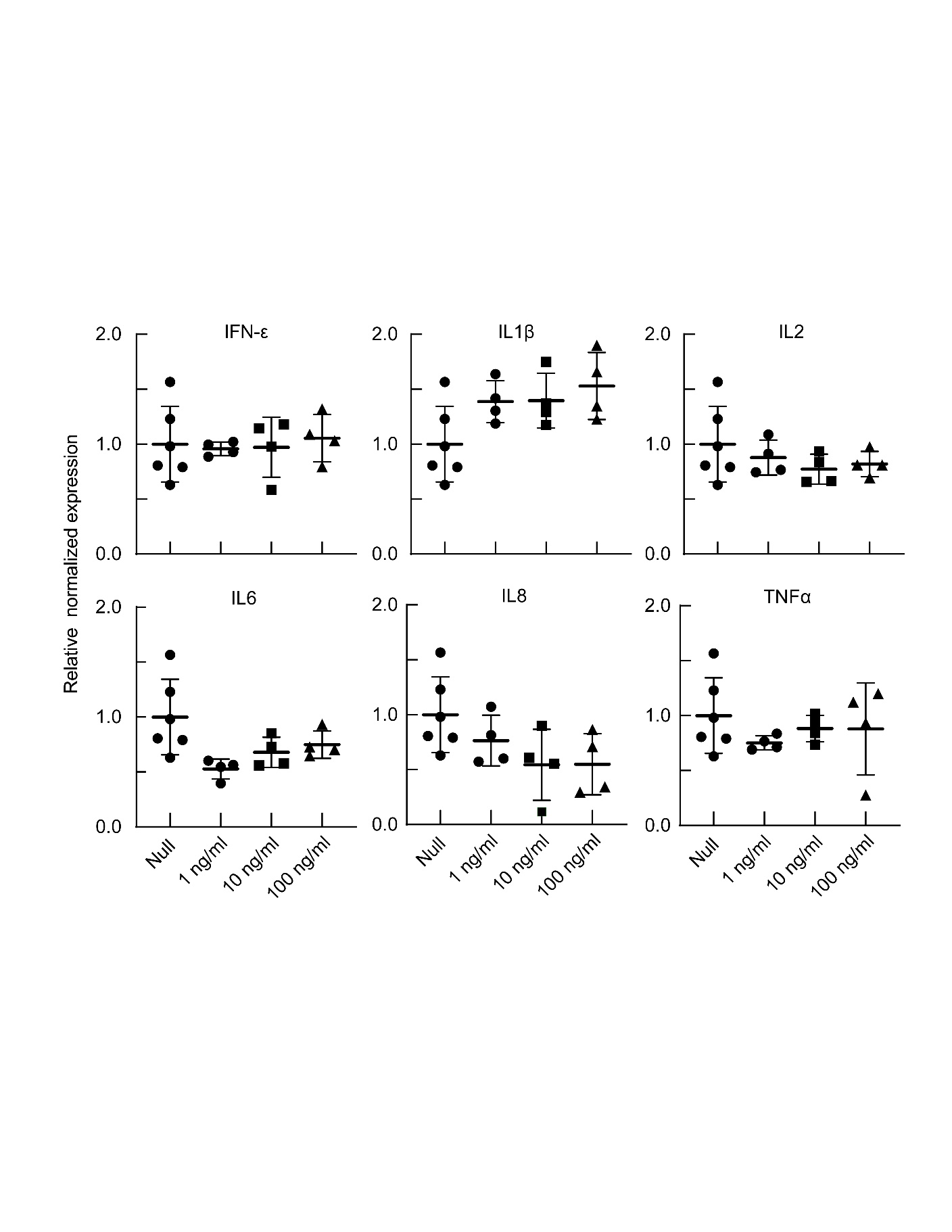


**Supplementary Fig. S6.** dd-PCR gene expression analysis of *Ace2* in HCEC after stimulation with 1, 10, and 100 ng/mL IFNε, IL1β, IL2, IL6, IL8, and TNFα. There was no significant increase in expression with any of the cytokines.


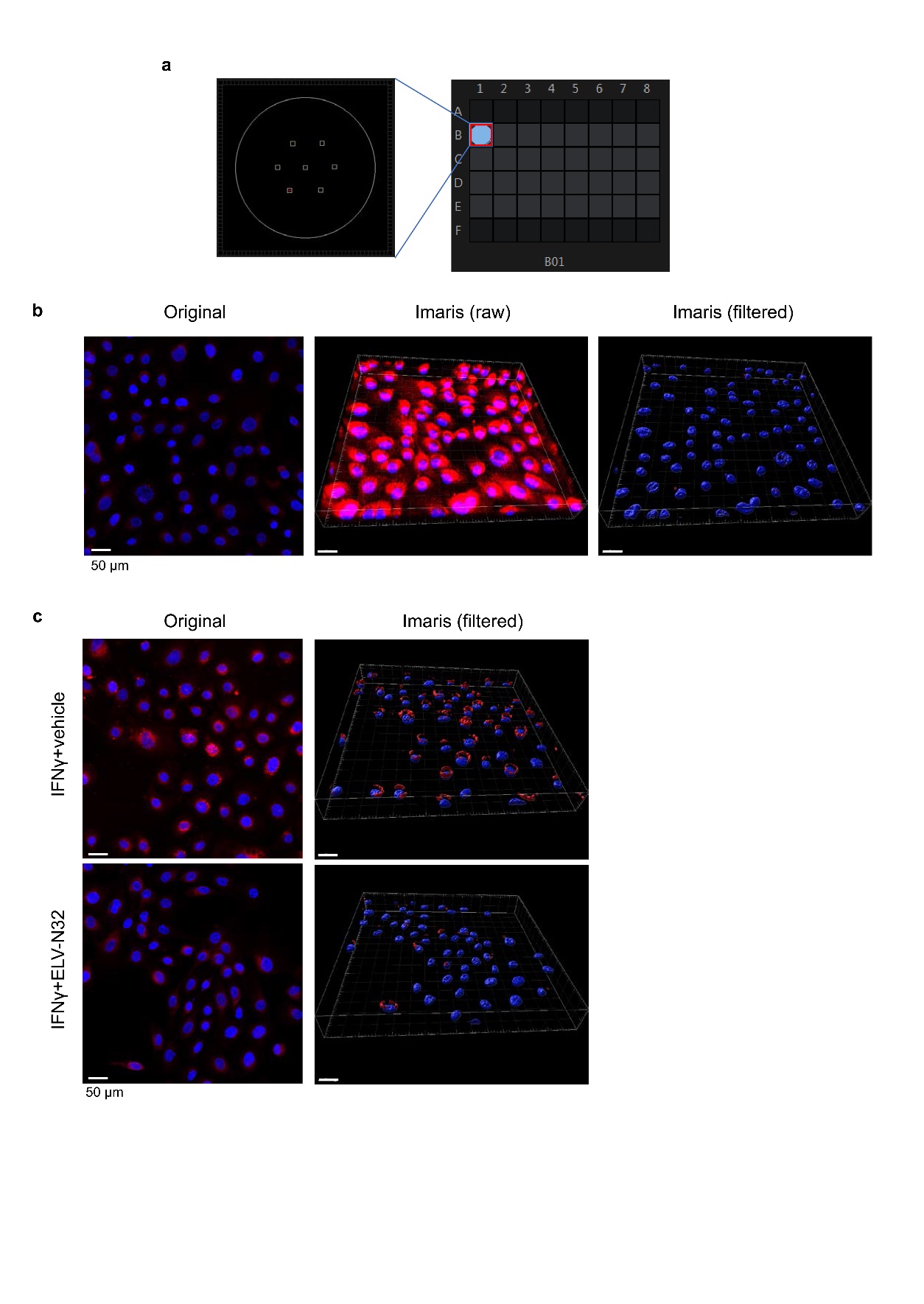
**Supplementary Fig. S7. a-b,** Unbiased imaging analysis of RBD binding in HCEC. **a**, Images were taken in the Multi Area Time Lapse mode with an Olympus FV3000 confocal microscope. For each well, 7 designed areas were taken with the same parameters and Z-section range. **b**, Images of a normal cornea showing the Imaris auto-fluorescence and the filtered image. All images were converted and inputted in the Imaris software, and the threshold for the control images (HCEC without Alexa 594-RBD) was defined. Then, the batch image processing was used to analyze all images with the defined threshold. The total sum intensity for each image was employed to evaluate binding efficiency. **c,** Representative images of Alexa 594-RBD for vehicle and ELV-N32 treated HCEC from the microscopy (left) and after Imaris threshold-filtration (right).


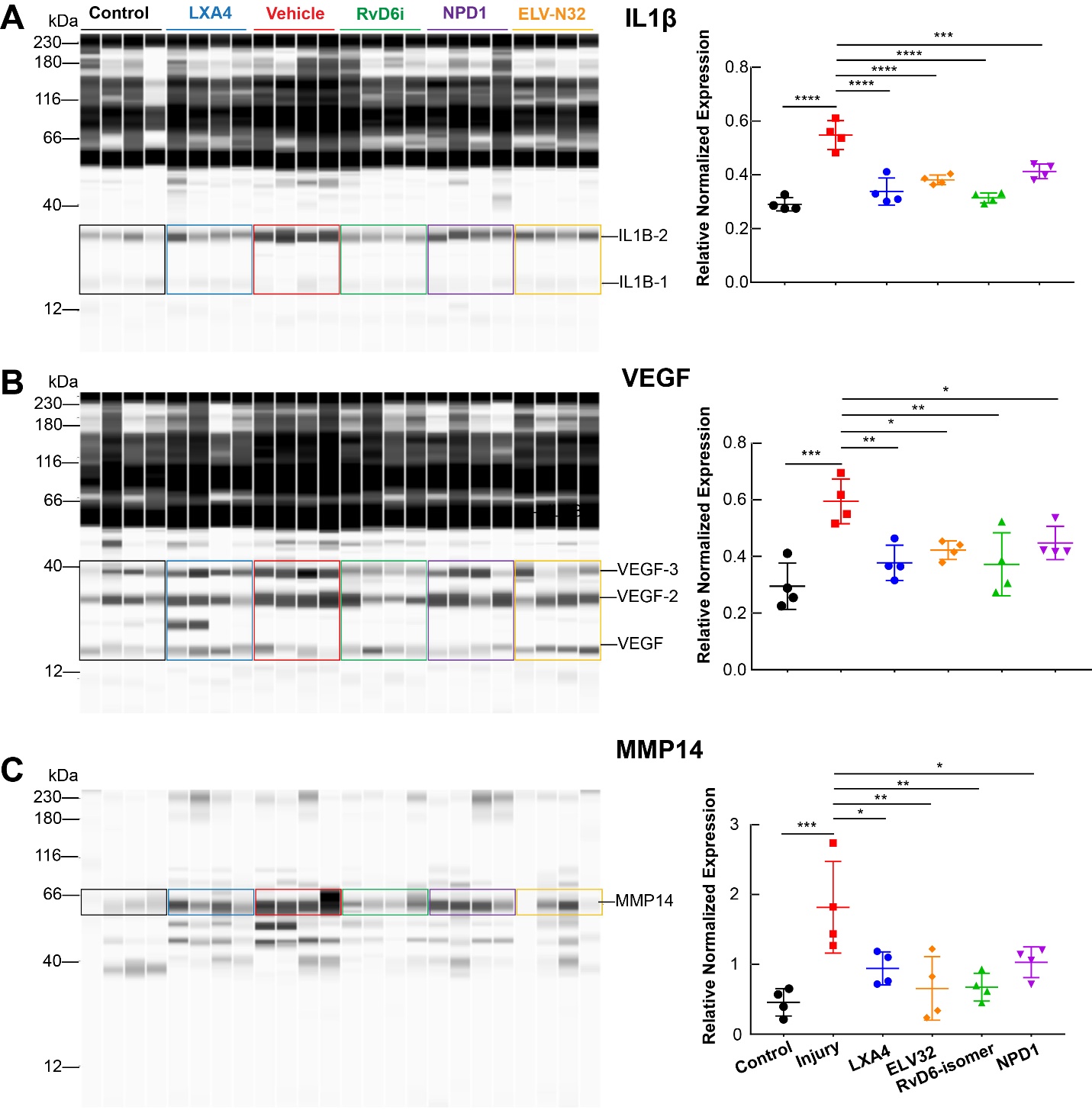


**Supplementary Fig. S8. a-d,** The Jess capillary-based western assay for semi-quantification of IL1β, VEGF, and MMP14 in the cornea before and after alkali burn and treatment. The left panels are the whole blot while the right panels are the quantification using GAPDH as reference.
